# Effects of Physical Exercise on Individual Resting State EEG Alpha Peak Frequency

**DOI:** 10.1155/2015/717312

**Published:** 2015-02-10

**Authors:** Boris Gutmann, Andreas Mierau, Thorben Hülsdünker, Carolin Hildebrand, Axel Przyklenk, Wildor Hollmann, Heiko Klaus Strüder

**Affiliations:** ^1^Institute of Movement and Neurosciences, German Sport University Cologne, 50933 Cologne, Germany; ^2^Institute for Cardiology and Sports Medicine, German Sport University Cologne, 50933 Cologne, Germany

## Abstract

Previous research has shown that both acute and chronic physical exercises can induce positive effects on brain function and this is associated with improvements in cognitive performance. However, the neurophysiological mechanisms underlying the beneficial effects of exercise on cognitive processing are not well understood. This study examined the effects of an acute bout of physical exercise as well as four weeks of exercise training on the individual resting state electroencephalographic (EEG) alpha peak frequency (iAPF), a neurophysiological marker of the individual's state of arousal and attention, in healthy young adults. The subjects completed a steady state exercise (SSE) protocol or an exhaustive exercise (EE) protocol, respectively, on two separate days. EEG activity was recorded for 2 min before exercise, immediately after exercise, and after 10 min of rest. All assessments were repeated following four weeks of exercise training to investigate whether an improvement in physical fitness modulates the resting state iAPF and/or the iAPF response to an acute bout of SSE and EE. The iAPF was significantly increased following EE (*P* = 0.012) but not following SSE. It is concluded that the iAPF is increased following intense exercise, indicating a higher level of arousal and preparedness for external input.

## 1. Introduction

Previous research has shown that both acute and chronic physical exercise can induce positive effects on brain function and this is associated with improvements in cognitive performance [1]. However, there is currently little understanding regarding neurophysiological mechanisms underlying the effects of exercise on cognitive processing and further investigation is required.

One approach to understand processes that underlie the beneficial effects of exercise on brain function and cognition has been to measure electrical brain activity using electroencephalography (EEG). Past research in this field focused on either a change in spectral power [2] and/or event-related potentials (ERPs) [3]. However, to the best of our knowledge, there are no studies examining the relationship between physical exercise and the individual alpha peak frequency (iAPF).

The iAPF corresponds to the discrete frequency showing the highest power within the alpha oscillation range (~7–13 Hz). It is considered a putative marker of an individual's state of arousal and attention [4], and it is positively related to the speed of information processing [5]. A number of studies have shown a positive relationship between iAPF and cognitive task performance. For example, individuals with higher iAPF show shorter reaction times [6], better working memory scores [7, 8], and superior memory performance [9]. In contrast, the iAPF is significantly reduced in Alzheimer's disease (AD), with the degree of the reduction being associated with the specific stage of AD [10]. Other diseases that are typically accompanied by cognitive decline are also characterized by a reduction of the iAPF. These include major depression [11], attention deficit hyperactivity disorder [12], cerebral ischemia [13], and carotid artery occlusion [14]. More recent studies identified that iAPF correlates with higher cerebral blood flow (CBF) in brain areas involved in attentional modulation and preparedness for external input [4]. In addition, subjects with higher iAPF show increased fractional anisotropy values in fascicles connecting networks of brain areas associated with working memory functions and attentional modulation [15].

Previous research showed that acute exercise increases CBF [16] and it was suggested that exercise activates arousal mechanisms within the brain, leading to improvements in implicit information processing [17]. Apart from these acute effects, aerobic exercise training has been shown to increase cerebral blood volume [18], perfusion [19], and the adult hippocampal volume [20]. In addition, a cross-sectional study revealed that aerobic fitness is positively associated with functional connectivity in the Default Mode Network, and this, in part, mediates better performance on tasks requiring set-shifting, task switching, and spatial working memory [21]. Consistent with this, one year of regular walking increased functional connectivity between parts of the frontal, posterior, and temporal cortices within the Default Mode Network and a Frontal Executive Network [22]. Moreover, greater aerobic fitness derived from a walking program was associated with increases in white matter integrity in the frontal and temporal lobes and greater improvement in memory performance [23].

Taken together, the above described literature suggests that physical exercise induces changes in the brain that should be associated with an increase in iAPF. Therefore, in a recent study, we analyzed the effect of an acute bout of exercise on the iAPF in young children [24]. Contrary to our hypothesis, the iAPF remained unchanged following exercise. One reason for this could be that exercise intensity was too low. Therefore, the aim of the present study was to investigate whether exercise intensity has an influence on the postexercise iAPF in healthy young adults. Moreover, a recent study suggests that even shorter term aerobic exercise training with minimal improvements in fitness can facilitate neuroplasticity in sedentary adults [25]. Therefore, another aim of this study was to explore the effects of a short term exercise training program on both baseline iAPF (before exercise) and the iAPF response to acute exercise. It was hypothesized that exercise intensity will have an influence on the effect of acute exercise on iAPF. Furthermore, baseline iAPF (before exercise) should be increased following exercise training and, thus, after training, the iAPF response to an acute bout of physical exercise may be attenuated.

## 2. Methods

10 male volunteers (age: 22.7 ± 2.0 years, body mass: 79.6 ± 6.9 kg, and height: 180 ± 4.8 cm) participated in the study. All of the subjects participated in regular exercise for the last two years (>30 min/day on >3 days/week) achieving current recommendations of the American College of Sports Medicine [26] prior to the study and were able to undertake exhaustive exercise. However, highly trained endurance athletes from sports such as long distance running, road cycling, and triathlon were excluded from the study. A preliminary medical assessment confirmed that participants were of good health and had no overt cardiovascular, orthopaedic, or neurological disorders. They were informed about the intention and procedure of the study and their written consent was obtained. The study was reviewed and approved by the Research Ethics Committee of the German Sport University Cologne in accordance with the Declaration of Helsinki.

**Figure 1:**
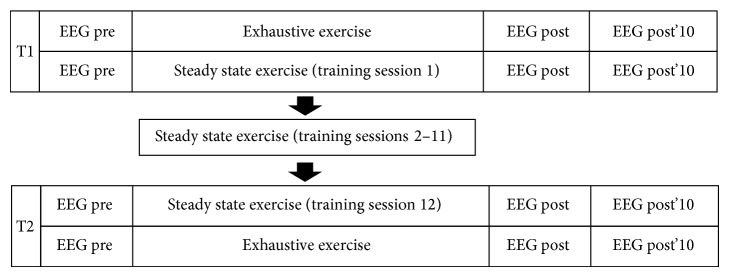
Schematic view of the experimental protocol.

On the first laboratory visit, participants were asked to adjust the seat and handlebars of the ergometer and to take a comfortable seated position. Individual ergometer settings obtained during this familiarization appointment were recorded and applied to all occasions in order to standardize the individuals' cycling position. The experimental protocol is depicted in Figure 1. There were two blocks of assessments: one before (T1) and one after four weeks of cycling training (T2). In each block, subjects were asked to complete an exhaustive exercise (EE) and a steady state exercise (SSE) protocol with one leg.

The EE was a ramp exercise protocol with initial intensity set at 30 W which was increased by 5 W every 30 sec until the subjects reached their point of exhaustion [27]. This protocol was conducted 36–48 h before and after the last training session in order to evaluate the effects of training on the subjects' maximum exercise capacity. All subjects completed three training sessions per week over a four-week period, resulting in a total of 12 training sessions. Each training session consisted of 30 min of one-legged cycling at 50% of the peak power output (PPO). The PPO of each leg was derived from a graded exercise protocol (starting at 40 W and increasing by 10 W every 3 min) on a separate occasion that allowed for determining the subjects' anaerobic lactate threshold. This was done to select an adequate training intensity slightly below the anaerobic lactate threshold to ensure it could be maintained for 30 min. This was equivalent to 65–75% of maximal heart rate (HR_max⁡_ = 220-age). The minimum intersession interval was set at 36 h to ensure adequate recovery. Cycling intensity was increased weekly by 5% to account for training effects. However, cycling intensity was identical in the first and the last training sessions to represent the SSE protocol before and after training, respectively.

At the end of each protocol, the subjects' heart rate (HR) (S810i, Polar Electro, Kempele, Finland) and rate of perceived exertion [28] were recorded. In addition, 20 *μ*L capillary blood was sampled from the earlobe to determine lactate concentration (BLa^−^) according to the enzymatic-amperometric principle of the Biosen C-Line (EKF Diagnostic, Barleben/Magdeburg, Germany).

Continuous resting state EEG was recorded for 2 min before exercise (pre), immediately after (post), and after 10 min of rest (post'10) (see Figure 1). During EEG recording (Brain Products GmbH, Munich, Germany) participants were asked to take a relaxed sitting position on the cycling ergometer, to close their eyes, and to avoid any movement. EEG was recorded from 15 scalp locations equally distributed over the scalp (Fp1, Fp2, F3, Fz, F4, T7, C3, Cz, C4, T8, P3, Pz, P4, O1, and O2) according to the international 10–20 system [29]. The electrodes were placed on a modular elastic cap with the electrical reference and ground electrode being located on positions FCz and AFz, respectively. Electrode impedances were kept below 5 kOhm. One electrooculographic (EOG) electrode was placed in horizontal line of the eyelid to identify eye movement artefacts. EEG data were sampled at 1000 Hz and were digitally band-pass filtered (high-pass 2 Hz, low-pass 120 Hz). The signal was rereferenced to the average potential of the 13 recording electrodes and segmented into epochs of 4 s. All epochs were baseline corrected and those epochs contaminated by eye or muscular artefacts were excluded from further analysis. Using fast Fourier transformation (FFT), the time domain data were transferred into power values in the frequency domain with a frequency resolution of 0.244 Hz. The iAPF was defined as the frequency bin showing the highest power value within 7–13 Hz at occipital sites (means O1 and O2). The occipital sites have been chosen according to numerous previous studies based on the rationale that alpha oscillations are the strongest over the parietooccipital cortical areas allowing reliable detection of the alpha peak [7, 30–32]. One subject was excluded from further analyses due to absence of a clear alpha peak.

Statistical comparisons of performance and physiological data, recorded after SSE and EE at T1 and T2, were performed using a repeated measurement analysis of variance (ANOVA) with the factors block (T1 versus T2) and protocol (EE versus SSE). The iAPF was analyzed calculating a repeated measurement analysis of variance (ANOVA) with the factors block (T1 versus T2), protocol (EE versus SSE), and time (pre versus post versus post'10). Mauchly's test was used to evaluate the sphericity assumption. In case of nonsphericity Greenhouse-Geisser correction was applied. Significant main effects and interactions were further analyzed using Fischer's LSD post hoc test.

## 3. Results

### 3.1. Exercise Parameters

Means and standard deviations (SD) of selected parameters recorded after the EE and SSE are summarized in Table 1. An ANOVA with the factors block (T1 versus T2) and protocol (EE versus SSE) was calculated for BLa^−^, HR, PPO, and RPE. As expected, a main effect for protocol was found for all parameters indicating higher values during EE when compared to SSE thus, confirming that EE was more intense than SSE (all *P* < 0.000). Furthermore, a significant block main effect for PPO (*P* < 0.000), as well as significant protocol × block interactions for BLa^−^, HR, and PPO, was observed. Subsequent post hoc analyses on the protocol × block interactions revealed significantly higher values for BLa^−^, HR, and PPO (all *P* < 0.05) after EE when compared to SSE at T1 and T2, respectively. In addition, PPO in the EE protocol increased by 13.95 ± 8.95% from T1 to T2 (*P* = 0.000). Finally, HR was significantly reduced and BLa^−^ showed a trend towards a reduction after SSE at T2 when compared to T1 (HR: *P* = 0.028; BLa^−^: *P* = 0.084). Together, these later results indicate an improvement in physical fitness following training.

### 3.2. Individual Alpha Peak Frequency

**Figure 2:**
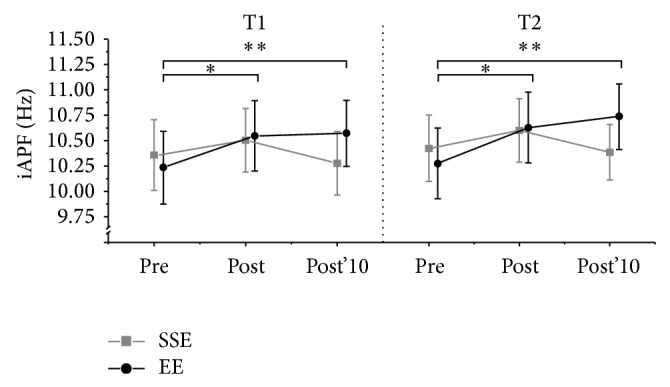
Mean individual alpha peak frequency (iAPF) before exercise (pre), immediately after exercise (post), and after 10 min of rest (post'10) following exercise. SSE: steady state exercise, EE: exhaustive exercise, T1: before training, and T2: after training. Error bars indicate standard error. Statistical differences: ^*^ *P* < 0.05; ^**^ *P* < 0.01.

Mean iAPF is depicted in Figure 2. An ANOVA with the factors block (T1 versus T2), protocol (EE versus SSE), and time (pre versus post versus post'10) yielded a significant main effect for time (*F*_2,16_ = 7.232, *P* = 0.006), as well as a significant protocol × time interaction (*F*_2,16_ = 3.995, *P* = 0.039). However, there were no significant effects for the factor block. Post hoc analysis on the protocol × time interaction revealed a significant increase in iAPF from pre to post (*P* = 0.012), as well as from pre to post'10 (*P* = 0.003), for the EE protocol. In contrast, the iAPF remained unchanged for the SSE protocol. Descriptive statistics of the iAPF are presented in Table 2.

## 4. Discussion

Although physical exercise has been shown to have positive effects on brain function and cognition across the lifespan [1], the underlying neurophysiological processes require further study. The present study examined the effects of an acute bout of physical exercise as well as four weeks of exercise training on the iAPF. The acute effect was examined following exercising at two different intensities, SSE and EE. The main finding is that the iAPF significantly increased immediately after EE and remained elevated for a minimum of 10 minutes. In contrast, the iAPF remained unchanged following SSE. Furthermore, four weeks of exercise training at steady state intensity did not result in any changes of the iAPF.

The iAPF is considered a putative marker of an individual's state of arousal and attention [4], as well as speed of information processing [33]. Therefore, the results of the present study indicate an acute bout of strenuous physical exercise activating mechanisms in the brain which facilitate information processing. This is in line with previous research on the effect of acute exercise on cognitive task performance. A recent metaregression analysis [34] indicated that, following exercise, cognitive task performance improved by a mean effect of 0.20. This improvement includes speeded mental processes, as well as memory storage and retrieval. In addition, some experiments measured young adults' sensory sensitivity before and after exhaustive cycling using the critical flicker fusion (CFF) frequency. CFF threshold and, thus, sensory sensitivity increased following exhausting exercise [35]. In contrast, steady state exercise induces an increase in CFF discrimination mainly during exercise, but it quickly returns to baseline levels immediately after exercise has ended [36]. In the same vein, reaction times (RT) are shorter during both maximal [37] and submaximal exercises [38]; however, the effect of submaximal exercise on RT disappears very quickly after exercise cessation [17, 39]. The results of the present study support previous research suggesting that acute exercise modulates arousal and activation, which remain temporarily elevated immediately after exercise. However, this effect depends on influencing variables such as intensity, duration, type of exercise, age, and the fitness level of the subjects as well as the time elapsed after exercise has ended [34].

It has been proposed that the arousing effect of acute exercise is mediated by changes in neural activity in the ascending reticular-activating system [40]. Within the brainstem, neurons of the reticular formation stimulate cortical activation by exciting the widespread projecting neurons of the nonspecific thalamocortical projection system [41]. These thalamocortical feedback loops of excitatory and inhibitory neurons are thought to be the primary generator of the alpha rhythm [42]. Therefore, the modulation of the iAPF following acute exercise is probably linked to exercise-induced activation of the brain's arousal mechanisms. In addition, acute exercise has been shown to increase CBF [16] and higher CBF correlated with iAPF in brain areas associated with working memory functions and modulation of attention [4, 15]. In sum, these results indicate that the increase in iAPF represents a potential neurophysiological mechanism underlying improvements in cognitive task performance following acute physical exercise.

The temporal characteristics of brain adaptations in response to exercise training are not well understood because only few intervention studies have been conducted so far. Previous aging studies focused largely on the effects of medium to long term (>6 months) exercise training programs, whereas the shorter term effects have not been studied until recently. Chapman et al. (2013) have shown that even shorter term (12 weeks) aerobic exercise training can facilitate neuroplasticity and promote brain health in sedentary adults [25]. Moreover, in the literature, changes in brain structure and function are often attributed to changes in fitness. Therefore, in the present study, we were interested whether a very short exercise training intervention (just enough to induce an improvement in physical fitness) is associated with a change in iAPF. The iAPF is functional measure, and therefore, adaptations may occur already after shorter training periods; before any structural changes take place. However, although exercise training for four weeks at steady state intensity was effective to induce an improvement in physical fitness, it was not sufficient to change iAPF. This is indicated by the absence of significant main effects and interactions for the factor BLOCK. This result suggests that an improvement in physical fitness does not necessarily imply neuroplasticity. It remains to be determined in future studies whether longer and/or more intensive periods of physical training may induce changes in iAPF.

Although it is well established that iAPF is correlated with cognitive performance, the proposed relationship between physical exercise, iAPF, and cognition in this study remains speculative as behavioral measures were not collected. We abstained from behavioral tests because cognitive engagement has also been shown to induce a transient increase in iAPF [43, 44]. Therefore, effects of physical exercise and cognitive engagement on iAPF could interfere. To avoid such interference, in a first step, we focused on the relationship between physical exercise and iAPF to provide a functional framework for future research such as addressing the significance of exercise-induced changes in iAPF on cognitive abilities in different populations (e.g., males and females, healthy and diseased, and young and old).

## 5. Conclusion

It is concluded that the iAPF, a neurophysiological marker for the individual's state of arousal and attention, increased following intense physical exercise. In contrast to intense physical exercise, the iAPF remained unchanged after 30 min of steady state exercise, and it is not altered following four weeks of steady state exercise training despite improvements in physical fitness. The cumulative pattern of results indicates a dose-response relationship between physical exercise and iAPF which needs to be further studied as it may have important implications for exercise recommendations to promote brain health and cognition.

## Figures and Tables

**Table 1:** Selected parameters for steady state exercise (SSE) and exhaustive exercise (EE) before (T1) and after (T2) training.

	**T1**				**T2**			
	Power (W)	Bla^−^ (mmol/L)	HR (1/min)	RPE (6–20)	Power (W)	Bla^−^ (mmol/L)	HR (1/min)	RPE (6–20)
	**SSE**							
Mean	74.52	3.73	141.05	12.33	74.52	2.88	131.50^a^	10.33
SD	9.03	1.07	13.73	1.38	9.03	0.77	10.12	2.05

	**EE**							
Mean	191.67^b^	8.40^b^	183.58^b^	20^b^	213.75^ab^	9.63^b^	185.60^b^	20^b^
SD	32.10	1.97	12.07	0	26.23	1.37	7.28	0

^a^Significant differences between T1 and T2 within SSE or EE; ^b^significant differences between the SSE and the EE protocol at T1 and T2, respectively. The significance level was set at *P* < 0.05. Bla^−^: blood lactate concentration, HR: heart rate, and RPE: Borg's rate of perceived exertion.

**Table 2:** Descriptive statistics of the individual alpha peak frequency (Hz) before exercise (pre), immediately after exercise (post), and after 10 min of rest (post'10) following exercise.

	**SSE**						**EE**					
	**T1**			**T2**			**T1**			**T2**		
	Pre	Post	Post'10	Pre	Post	Post'10	Pre	Post	Post'10	Pre	Post	Post'10
Mean	10.36	10.51	10.28	10.42	10.60	10.38	10.23	10.55^*^	10.57^**^	10.28	10.63^*^	10.74^**^
SD	1.32	1.20	1.03	1.02	1.12	1.07	1.19	1.20	1.13	1.25	1.22	1.15

SSE: steady state exercise, EE: exhaustive exercise, T1: before exercise training, and T2: after exercise training. Statistical differences: ^*^ *P* < 0.05; ^**^ *P* < 0.01.
